# Parent and Patient Satisfaction after Treatment for Supracondylar Humerus Fractures in 139 Children: No Difference between Skeletal Traction and Crossed Pin Fixation at Long-Term Followup

**DOI:** 10.1155/2012/958487

**Published:** 2012-02-19

**Authors:** Sven Young, Jonas M. Fevang, Gunnar Gullaksen, Per T. Nilsen, Lars B. Engesæter

**Affiliations:** ^1^Department of Orthopaedic Surgery, Haukeland University Hospital, 5021 Bergen, Norway; ^2^Department of Orthopaedic Surgery, Akershus University Hospital, 1478 Lørenskog, Norway

## Abstract

*Aim*. The aim of this study was to see whether the benefits of crossed wire fixation over skeletal traction in the treatment of pediatric supracondylar humerus fractures (SCHF) were mirrored in the children's or their caregivers' rating of the experience. *Methods*. As part of a study of the clinical outcome of SCHF, all the patients and the parents were asked to rate their experience of the treatment on a visual analogue scale (VAS). *Results*. There was no difference in the patients' or the parents' experience between the treatment groups. However there was a difference between the parents with children who experienced a neurovascular complication (mean VAS 6.1) and those that did not (mean VAS 4.3, *P* = 0.03). The boys rated the experience as less negative (mean VAS 3.6) than the girls (mean VAS 4.7, *P* = 0.02). *Conclusion*. In the long term, avoiding complications was more important to the parents than the choice of treatment for SCHF in the children.

## 1. Introduction

Supracondylar humerus fractures (SCHFs) are the fractures in childhood most associated with serious complications [1–8]. Undisplaced fractures, Gartland type 1, are generally treated with immobilization in a cast with good results [6, 9, 10]. However, there has been a considerable danger of serious complications with the treatment of displaced SCHF in a plaster of Paris (POP) cast with hyperflexion [8]. For this reason Dunlop used straight arm skin traction for these fractures from the late nineteen twenties [11]. The introduction of traction reduced the number of complications associated with this fracture [12], and skeletal traction with a K-wire [9, 13] or a screw [14] in the olecranon has later shown to provide better control of fracture reduction. From the nineteen thirties and onward, several papers were published on K-wire fixation of SCHF [15–17], until this became the treatment of choice for this fracture in children in most hospitals in Europe and America during the nineteen eighties and nineties. It was introduced to Haukeland University Hospital (HUH) in 1992 by the senior author (L. B. Engesaeter). Prior to this, overhead skeletal traction was used as the standard treatment for SCHF at HUH. From 1992 until crossed wire fixation was firmly established as the treatment of choice in 1995, skeletal traction was still used by some surgeons on call [18]. HUH is the tertiary referral centre for pediatric orthopedics and trauma for Western Norway and serves a population of approximately 900 000. It is the only hospital offering treatment for displaced supracondylar humerus fractures in a population of approximately 300 000 in and around the city of Bergen. The majority of undisplaced fractures are treated at a separate institution.

Though there are obvious and well-proven benefits of wire fixation over skeletal traction, such as a shorter hospital stay [18], to our knowledge, no studies of patient and caregiver satisfaction comparing different treatments for SCHF exist. The aim of this study was to investigate whether the presumed benefits of crossed wire fixation over skeletal traction are mirrored in the children's or their caregivers' subjective experience of the treatment at long-term followup.

## 2. Patients and Methods

268 children were treated at the Haukeland University Hospital for a pediatric distal humerus fracture from 01.01.1988 to 31.12.1998. On review of the case notes of all these patients, 181 consecutive patients with isolated supracondylar humerus fracture were identified. These were invited for a follow-up visit to the pediatric orthopedic outpatient clinic at HUH. 141 of the 181 patients (78%) attended the clinic. Two of these on closer examination turned out to be lateral humerus condyle fractures and were excluded, leaving 139 patients for this study. The parents of all children gave their informed consent to themselves and their child taking part in this study. The epidemiological and clinical results have been published in an earlier article [18].

The parents/caregivers and the children were all asked to rate their experience of the fracture and its treatment on a visual analogue scale (VAS) from 0 to 10 where 0 was “No problem at all” and 10 was “A terrible experience.” The children were examined with regards to elbow function, neurovascular function, and deformity. The medical records of all the patients were examined separately from the clinical examination. We recorded the injured arm, the date and time of injury, time of admittance, and time of surgery. Pre- and postoperative vascular status, nerve function, infection, and number of reoperations were also noted. VAS scores were compared according to fracture classification, four different treatment groups, the presence or absence of a complication, the time from injury to surgery, and the time from injury to followup.

### 2.1. Fracture Classification

The fractures were classified retrospectively at followup using Wilkins' modified version of Gartland's classification [6, 10].

### 2.2. Treatment Groups

The patients were divided into four groups according to the treatment they received; POP cast only (40 children), overhead skeletal traction (46 children), closed reduction with percutaneous crossed wire fixation (45 children), and open reduction with crossed wire fixation (8 children) [18]. The undisplaced fractures were treated with a plaster of Paris (POP) cast with the elbow flexed at 90 degrees. Overhead skeletal traction was performed using a winged Palmer olecranon screw. After a mean 11 days in traction a POP, cast was applied and the Palmer screw was removed, usually in general anesthesia (GA). Crossed wire fixation was done percutaneously and a cast applied in GA. The pins were removed in the outpatient department after four weeks with only acetaminophen as premedication. Eight patients were treated with open reduction and crossed pin fixation because of failure to reduce the fracture by closed means or due to vascular injury.

### 2.3. Complication Group

Seventeen (12%) of the 139 children had an observed nerve injury at some point during treatment or followup. 15 of these had Gartland type 3 fractures. No nerve injuries were recorded in patients with Gartland type 2 fractures. In the plaster cast group, two children with fractures classified as Garden type 1 were found to have sensory loss in the distribution of the median and radial nerve, respectively, at the followup. One child had a cold, pale, and pulseless arm necessitating exploration and repair of the brachial artery. Three other children had an absent radial pulse that later normalized. All the 21 (15%) children that experienced a complication were defined as one group for the detailed analysis.

### 2.4. Statistics

The Student *t*-test was used to compare the means of the continuous variables between the treatment groups. All *P* values were two tailed, and the level of statistical significance was set to 5% (*P* ≤ 0.05). The data was analyzed using SPSS version 17.0 (SPSS Inc., Chicago, IL 60606, USA). Power analysis was carried out using G*Power 3.1.2 (Faul, Erdfelder, Lang, and Buchner, 2006, 2009) [19]. It showed that (given sample sizes of 45 patients, *α* = 0.05, and a power of 80%) measurable effect size would be approximately 0.6. This would, in our material, be equivalent to approximately a difference in VAS of 1.7.

## 3. Results

**Figure 1:**
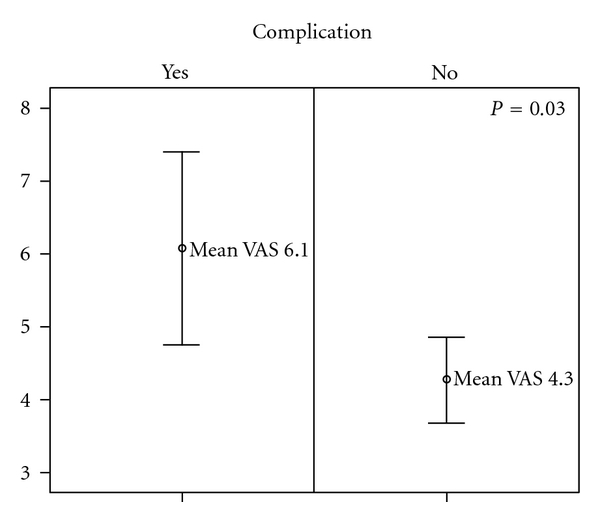
Parents' rating of their experience of their child's SCHF on a visual analogue scale (VAS) according to if the child experienced complications to the fracture or not. VAS 0 = “no problem at all”, 10 = “a terrible experience.” Mean VAS ± 2 standard error of the mean (SEM).

A total of 104 parents and 111 children rated their experience on a predesigned VAS. The results with mean VAS for each group, along with the number of cases and the standard error of the mean (SEM), are presented in Table 1. The mean time from injury to followup over all was 7.1 years (SEM 0.27), 5.2 years (SEM 0.36) for the crossed pin fixation group, and 9.9 years (SEM 0.38) for the skeletal traction group (*P* < 0.001). In the skeletal traction group mean age at followup was 16.7 (SD 3.8) years. The mean age in the wire fixation group was 11.8 (SD 3.0) years at followup. Parents rated their experience of their child being treated for SCHF on average as 4.5 (SEM 0.27) on a VAS scale from 0 to 10. The children's mean VAS score was 4.1 (SEM 0.24, *P* = 0.22). There was no statistical difference in the children's or the parents' VAS scores between the POP, skeletal traction, or crossed wire fixation groups. Likewise, there was no statistical difference in VAS scores according to the severity of the fracture (Gartland's type), time from injury to surgery more or less than eight hours or time passed from injury to followup. However, there was a significant difference (*P* = 0.03, Figure 1) between a more negative experience by the parents who had experienced a neurovascular complication in their children at some time during the treatment (mean VAS 6.1) and those parents who did not (mean VAS 4.3). The same trend (*P* = 0.10) could be observed in the equivalent groups of children. There was also a difference (*P* = 0.02) in how the boys (mean VAS 3.6) and the girls (mean VAS 4.7) perceived their experience, whereas their parents did not differentiate between the girls (mean VAS 4.5) and the boys (mean VAS 4.5).

## 4. Discussion

The VAS is a quick and easy method of rating a subjective experience such as pain and anxiety and has been used in similar studies both with children and adults [20, 21]. The use of a single VAS score to rate patients' and caregivers' experience with a treatment may not give a complete picture of this experience, but in our opinion gives an idea of how positive or negative that experience was.

The main findings in our previous article describing the clinical and epidemiological findings in these patients [18] were that the introduction of crossed wire fixation reduced the median length of stay (LOS) from 11 to 2 days and that there was no statistically significant difference in complication rates between these two treatment groups. The large reduction in LOS was intuitively an obvious improvement for the children and their parents. There were also more reoperations in the skeletal traction group (4/46) compared to crossed wire fixation (0/45, *P* = 0.04) because of unsatisfactory reduction of the fracture. Given the nature of skeletal traction treatment, the fact that the children needed to be hospitalized more than ten days, and the need to undergo general anesthesia twice, we had expected to find that crossed wire fixation was less of a traumatic experience than skeletal traction. However, this was not the case in this study. No statistically significant differences could be found in any of the children's VAS scores. In light of this one must ask one's self if a child's rating of such an experience several years after it happened can be trusted. There was a slight tendency for the children's VAS scores (mean 4.05) to be lower than their caregivers' scores (mean 4.5). This would support the general feeling that the children “forgive and forget” faster than their parents. However, the difference was not statistically significant (*P* = 0.22) with the current study size and design. One could, along the same lines, argue that the relatively long followup was too long and that it would be expected that the mean VAS score would decrease proportionately to the number of years gone by since the fracture, but, as can be seen from Table 1, this does not appear to be the case either. Time to followup in the wire fixation and traction groups was different, and this difference in the children's maturity, or time gone by since the fracture, might be a source of bias. 

The fact that the boys in this series rated their experience less severe than the girls could, of course, be due to the boys' not wanting to admit it was a bad experience as well as an actual difference in their perception of the experience. Either way it did display a gender difference we did not expect to find. It was also interesting to see that their parents did not differentiate between the girls and the boys.

The present study has the usual limitations of being a retrospective study when it comes to collection of data about the fractures and early complications. However, the information on the children's and their caregivers' experience of the treatment was prospectively collected as part of a dedicated study out patient visit. In our opinion, the fact that the patients were included consecutively before and after the introduction of a new method, with as little separation in time as possible, makes the groups comparable. One could argue that there was a selection bias as only 111 children and 104 parents rated their experience of treatment for SCHF. However, of the 181 patients identified from the hospital files as having been treated for SCHF and invited to participate in the study, 78% consented and returned for followup. The relatively lower number of parents that responded can be explained by the fact that not all the children came for followup with their parents. Some of them came alone, with older siblings, their grandparents, or another guardian. Also, not all the children could or wanted to rate their experience. All this considered, we do not think there is reason to believe there has been a large selection bias. Power analysis showed the sample size to be sufficiently large to detect clinically significant differences, though smaller differences could be overlooked. The statistically significant difference in VAS between parents with children who experienced a neurovascular complication at some time during the treatment and those that did not is hardly surprising. However, in our opinion, the study has shown that the parents' VAS ratings were not completely random, even after five to ten years. In effect, it is likely that the findings that there are no differences in their experience of the different treatment methods at long-term followup can be trusted.

There was no difference in the children's or the caregivers' negative experience at long-term followup after treatment for supracondylar humerus fractures as rated on a visual analogue scale when comparing POP, skeletal traction, or crossed wire fixation. Caregivers to children that experienced a neurovascular complication, however, had a more negative experience independent of treatment or fracture type. In the long term, then, whether or not complications could have been avoided seems to be more important to the caregivers than the choice of treatment for supracondylar humerus fractures in the children. Though this might be of mostly historical interest in high-income countries, in a limited resource setting—where traction and casting often still is the only treatment option for these injuries—surgeons may use this information to inform parents and to prioritize the use of resources in an orthopaedic department.

## Figures and Tables

**Table 1:** Parents' and children's rating of experience of being treated for SCHF. VAS scale 0–10. 0 is no negative experience at all, 10 a very negative experience.

	Treatment							

	POP		Skeletal traction		Closed reduction and crossed pin fixation		Open reduction and crossed pin fixation	
	VAS (*n*)	SEM	VAS (*n*)	SEM	VAS (*n*)	SEM	VAS (*n*)	SEM

Parents	4.1 (32)	0.48	4.8 (24)	0.50	4.7 (42)	0.47	4.2 (6)	1.1
Children	3.4 (30)	0.43	4.4 (40)	0.37	4.4 (35)	0.46	3.5 (6)	1.4

	Fracture dislocation							

	Gartland's type 1		Gartland's type 2		Gartland's type 3			
	VAS (*n*)	SEM	VAS (*n*)	SEM	VAS (*n*)		SEM	

Parents	4.3 (15)	0.75	4.2 (30)	0.46	4.6 (56)		0.38	
Children	3.5 (17)	0.44	3.4 (24)	0.51	4.4 (66)		0.33	

	Time from injury to followup							

	Followup 0–4 years		Followup 5–7 years		Followup ≥8 years			
	VAS (*n*)	SEM	VAS (*n*)	SEM	VAS (*n*)		SEM	

Parents	4.1 (42)	0.45	4.6 (35)	0.48	4.9 (26)		0.49	
Children	3.7 (37)	0.42	4.4 (26)	0.52	4.1 (47)		0.36	

	Time from injury to surgery							

	< 8 hours				≥8 hours			
	VAS (*n*)		SEM		VAS (*n*)		SEM	

Parents	4.3 (47)		0.39		3.4 (15)		0.62	
Children	4.3 (45)		0.41		2.7 (12)		0.60	

	Gender							

	Girls				Boys			
	VAS (*n*)		SEM		VAS (*n*)		SEM	

Parents	4.5 (45)		0.38		4.5 (59)		0.38	
Children	4.7* (46)		0.38		3.6* (65)		0.30	

	Experience of neurovascular complications							

	Complications				No complications			
	VAS (*n*)		SEM		VAS (*n*)		SEM	

Parents	6.1** (13)		0.67		4.3** (91)		0.29	
Children	4.9*** (18)		0.62		3.9*** (93)		0.26	

*P = 0.02, **P = 0.03, ***P = 0.10.

SEM = standard error of the mean.
